# Anaphylaxis Secondary to Aeroallergen Immunotherapy in a Military Member

**DOI:** 10.7759/cureus.95954

**Published:** 2025-11-02

**Authors:** Ava P Klein, Jackson L Howell, Christopher Coop, Brittanie Neaves

**Affiliations:** 1 Perioperative Unit, Keesler Medical Center, Biloxi, USA; 2 Allergy and Immunology, Keesler Medical Center, Biloxi, USA

**Keywords:** active-duty military personnel, allergy and anaphylaxis, allergy immunotherapy, deployment medicine, military medicine, military readiness

## Abstract

We present the case of a 43-year-old active-duty US Air Force service member who experienced an anaphylactic reaction during routine aeroallergen immunotherapy. The patient had a long-standing history of perennial and seasonal allergic rhinoconjunctivitis, with confirmed sensitization to multiple environmental allergens through skin prick and serum-specific IgE testing. He tolerated build-up immunotherapy until reaching the 0.4 mL injections from the maintenance 1:1 volume/volume (1:100 weight/volume) vials, at which point he developed an anaphylactic reaction characterized by vomiting, hypotension, and respiratory distress. He required three intramuscular doses of epinephrine and was transferred to the emergency department. This case underscores the complexities of allergy management in military personnel, particularly those receiving immunotherapy in preparation for deployment. The patient’s severe systemic reaction raises important considerations regarding risk stratification, protocol modification, and implications for operational readiness. Although he was deemed fit for duty after stabilization, this event highlights how environmental allergen exposure and immunotherapy-related complications can impact force health protection. Proactive allergy screening, patient education, and emergency preparedness, especially ensuring access to and proper use of epinephrine auto-injectors, remain essential components of pre-deployment medical planning. This case reinforces the importance of individualized immunotherapy protocols, vigilant monitoring for systemic reactions, and comprehensive contingency planning within military healthcare systems.

## Introduction

Allergic rhinoconjunctivitis is a common atopic condition affecting 20%-40% of adults in the United States and often leads to significant impairment in the quality of life, especially in active military personnel exposed to challenging environmental conditions [1,2]. Aeroallergen immunotherapy remains a cornerstone of treatment for individuals with moderate-to-severe environmental allergies, offering the potential for long-term desensitization. However, systemic reactions, including anaphylaxis, can happen in rare cases and may require emergency intervention. While most immunotherapy reactions are mild, patients with extensive sensitization, comorbid illness, or active viral infections may be at higher risk for severe responses [3]. In military settings, the safe administration of allergy immunotherapy is further complicated by operational demands, geographic variability in allergen exposure, and limited access to emergency care in deployed environments [4]. We present the case of a service member who developed severe anaphylaxis during buildup-phase immunotherapy, reinforcing the importance of risk stratification, environmental control strategies, and pre-deployment allergy management.

## Case presentation

A 43-year-old US Air Force male officer with a history of perennial and seasonal allergic rhinoconjunctivitis presented for continued allergy care after experiencing a severe systemic reaction during his routine aeroallergen immunotherapy. The patient had no prior history of systemic reactions, no asthma symptoms, and no known drug or food allergies (NKDA). Additionally, he was not on any beta-blocker medications. His previous symptoms primarily involved nasal congestion, eye itching, rhinorrhea, and sneezing, triggered by dust, pollen, and grass exposure. 

Skin prick testing (Table 1) revealed strong reactivity to multiple environmental allergens, including Bahia and Bermuda grasses (wheal 20/15 mm, flare 40/22 mm), and dust mites *Dermatophagoides farinae* and *Dermatophagoides pteronyssinus* (wheal 8-11 mm, flare up to 32 mm) with positive histamine control (wheal 3 mm, flare 3 mm) and no response to negative control.

**Table 1:** Skin prick testing results "+" positive result; "-" negative result.

	Wheal	Flair	(+/-)
1 Histamine control	3	3	+
Trees			
2 Ash, white	2	3	-
3 Beech, American	0	0	-
4 Birch mix (RWB)	0	0	-
5 Box elder	0	0	-
6 Cedar, mountain	3	5	+
7 Cottonwood, common	0	0	-
8 Cypress, bald	0	0	-
9 Maple, red	0	0	-
10 Gum, sweet	3	3	+
11 Hackberry	0	0	-
12 Mulberry, red	0	0	-
13 Oak mix	12	35	+
14 Pecan	0	0	-
15 Pine mix	3	16	+
16 European olive	0	0	-
17 Sycamore, east	1	1	-
18 Walnut, black	3	4	+
19 Willow, black	4	15	+
Grasses			
20 Bahia	20	40	+
21 Bermuda	15	26	+
22 Johnson	0	0	-
23 June, ky blue	0	0	-
24 Rye, perennial	0	0	-
25 Timothy	0	0	-
Environmentals			
26 Cat, standard	7	30	+
27 Dog, UF	0	0	-
28 Cockroach mix	0	0	-
29 Mite, *Dermatophagoides farinae*	0	0	-
30 Mite, *Dermatophagoides pteronyssinus*	11	32	+
Weeds			
31 Dock/sorrel mix	15	26	+
32 Burning bush (Kochia)	0	0	-
33 Lambs quarter	0	0	-
34 Marshelder, rough	0	0	-
35 Nettle	0	0	-
36 Pigweed mix (CPR)	7	30	+
37 Plantain, English	0	0	-
38 Ragweed mix	0	0	-
39 Russian thistle	0	0	-
40 Sage, mugwort	11	32	+
41 Cocklebur	2	14	-
Molds			
42 *Alternaria*	2	4	-
43 *Aspergillus fumigatus*	2	4	-
44 *Drechslera *(*Curvularia*)	0	0	-
45 *Epicoccum*	0	0	-
46 *Helminthosporium*	3	3	+
47 *Hormodendrum*	0	0	-
48 Mucor mix	4	4	+
49 *Penicillium*	0	0	-
50 Negative control	0	0	-

Serum-specific IgE testing (Table 2) confirmed sensitization to several grasses and trees, including common ragweed (18.40 kU/L), Bermuda grass (15.60 kU/L), Timothy grass (41.00 kU/L), oak (5.85 kU/L), elm (4.48 kU/L), and mugwort (2.75 kU/L), as well as *D. pteronyssinus* (11.60 kU/L) and *D. farinae* (6.42 kU/L). His baseline tryptase level was elevated at 60.7 ng/mL, supporting the diagnosis of systemic anaphylaxis.

**Table 2:** Serum-specific IgE testing results Note: Anything greater than 0.10 kU/L is indicative of allergen-specific IgE sensitization. IgE units were calculated using blood sample analysis and enzyme-linked immunosorbent assay.

Lab test	Result
Wheat, Ab IgE	1.70 kU/L
Soybean, Ab IgE	0.77 kU/L
Rye food, Ab IgE	1.20 kU/L
Rice, Ab IgE	0.71 kU/L
Potato, Ab IgE	2.01 kU/L
Peanut, Ab IgE	1.66 kU/L
Green pea, Ab IgE	0.53 kU/L
Oat, Ab IgE	0.91 kU/L
Milk, Ab IgE	0.79 kU/L
Egg yolk, Ab IgE	<0.35
Egg white, Ab IgE	0.58 kU/L
Corn, Ab IgE	0.91 kU/L
Chicken meat, Ab IgE	<0.35
Beef, Ab IgE	<0.35
Barley, Ab IgE	1.02 kU/L
Timothy grass, Ab IgE	41.00 kU/L
Russian thistle, Ab IgE, RAST class	2.61 kU/L
Common ragweed, Ab IgE, RAST class	18.40 kU/L
Oak, Ab IgE	5.85 kU/L
Mugwort, Ab IgE, RAST class	2.75 kU/L
Lambs quarter, Ab IgE	2.33 kU/L
Juniper mountain, Ab IgE, RAST class	3.82 kU/L
Johnson grass, Ab IgE	20.70 kU/L
Bermuda grass, Ab IgE	15.60 kU/L
Elm, Ab IgE	4.48 kU/L
Dog dander, Ab IgE	0.96 kU/L
*Dermatophagoides pteronyssinus*, Ab IgE	22.10 kU/L
*Dermatophagoides farinae*, Ab IgE	21.20 kU/L
Cat dander, Ab IgE	0.55 kU/L
*Cladosporium herbarum*, Ab IgE	<0.35
Birch, Ab IgE	4.46 kU/L
White ash, Ab IgE	6.30 kU/L
*Aspergillus fumigatus,* Ab IgE	<0.35
*Alternaria alternata,* Ab IgE	<0.35

On October 29, 2024, during a scheduled immunotherapy visit, he received 0.4 mL of 1:1 volume/volume dilution (red vial). Within two minutes, he developed flushing, conjunctivitis, nausea, vomiting, dizziness, presyncope, and respiratory distress. He was administered three intramuscular injections of 0.3 mg epinephrine (two in the left leg, one in the right leg), given 6 L oxygen via face mask, and laid supine in the Trendelenburg position. He became hypotensive with his blood pressure dropping to 105/65 mmHg, his heart rate rose to 110 bpm, and his oxygen saturation fell to 89%. He was subsequently transferred to the emergency room for further observation and management. His tryptase level drawn at the emergency room was 60.7 µg/L. No oral medications were administered during the episode. He takes cetirizine 10 mg daily to twice daily for his allergic rhinoconjunctivitis symptoms, helping to manage his post-interventional clinical condition. 

Despite the severity of his reaction, the patient had no history of asthma, cardiovascular disease, or prior anaphylaxis. However, he reported mild viral upper respiratory symptoms in the week prior to injection, which he had not disclosed on the health screener and which may have contributed to heightened reactivity. The patient reported minimal memory of the incident beyond the first dose of epinephrine. Because of his severe anaphylactic reaction to immunotherapy, he elected not to restart aeroallergen immunotherapy. 

## Discussion

This case illustrates a severe, unexpected anaphylactic reaction to subcutaneous aeroallergen immunotherapy in a previously tolerant patient, emphasizing the potential volatility of immune responses in highly sensitized individuals. The elevated serum tryptase level further supports the diagnosis of mast cell-mediated anaphylaxis.

In the context of military operations, allergic diseases like rhinoconjunctivitis are not typically disqualifying; however, the risk profile changes when anaphylactic reactions occur. The US Department of Defense (DoD) maintains that any condition interfering with a service member’s ability to safely perform duties, especially under austere conditions, must be assessed for deployability [5,6]. In this case, the patient's condition raises several readiness considerations. Due to the austere environments military members may experience, field limitations play a part in the overall case outlook. In deployed environments, access to medical care, epinephrine, and climate-controlled environments can be limited. Mask and gear interference may play a role as well. Environmental allergies can interfere with the seal and tolerability of gas masks, helmets, and other protective equipment, impairing performance and mission execution [7]. An anaphylactic reaction without immediate access to care could result in morbidity or mortality. Epinephrine requires specific temperature conditions (15-30°C). High heat or humidity could degrade the medication's efficacy, rendering the only line of defense ineffective in combat environments [8]. Additionally, the potential loss of operational capacity plays a role in management decisions when looking at personnel decisions. Rhinoconjunctivitis can potentially impact mission readiness even in less serious cases. Eye irritation, fatigue, breathing issues, and other common symptoms all play a role in readiness. These symptoms can be exacerbated in outdoor field operations [7]. In more serious cases, the potential for unscheduled medical evacuations or inability to participate in field activities poses a risk to unit cohesion and mission success. Service members are also potentially exposed to multiple sources of air pollutants. In recent deployments, these pollutants have ranged from dust particulates, burn pit products, and exposure to the burning of waste in open-air, and pollutants from other military-related activities. This results in many immediate and long-term respiratory conditions, such as worsened cases of asthma and rhinitis, pulmonary infections, and chronic obstructive pulmonary disease. This adds an interesting outlook to the case of individuals with allergic rhinoconjunctivitis, as these air pollutants can exacerbate symptoms and potentially cause other respiratory conditions that will make overall respiratory function worse [9]. 

Despite these concerns, the patient demonstrated adherence to avoidance strategies, understanding of his condition, and competence in emergency response protocols. These qualities supported the conclusion that he remained medically deployable with appropriate precautions, including carrying dual epinephrine auto-injectors, strict compliance with environmental control measures, and allergic rhinoconjunctivitis medications.

## Conclusions

This case demonstrates the complexity of managing allergic diseases in active-duty military personnel, particularly when severe reactions occur in otherwise manageable conditions. While immunotherapy is generally safe and effective, even well-controlled patients can experience unpredictable systemic events. For military personnel, these risks extend beyond individual health and have implications for unit readiness, emergency planning, and healthcare logistics.

A proactive approach, including allergen-specific testing, personalized immunotherapy schedules, patient education, and medical contingency planning, can help mitigate risks. Service members with known allergic conditions should undergo thorough screening before deployment, be equipped with self-injectable epinephrine, and be evaluated for compatibility with field environments. This case highlights the need for DoD allergy programs to strike a balance between therapeutic benefits and operational safety, emphasizing individualized care while maintaining military effectiveness.
